# PEM as an adjunct to MRI or CESM for characterizing non-mass enhancement: a retrospective two-arm diagnostic study

**DOI:** 10.1186/s40644-026-01046-4

**Published:** 2026-05-13

**Authors:** Nourhan Emad El-Din Ali Mohamed, Dalia Elmesidy, Rasha Wessam Abdel Rahman, Abdel Rahman Omar Ahmed, Maher Hassan Ibraheem, Ghada Mohamed Abdel Salam, Aalaa Sobhi

**Affiliations:** 1 Department of Radiology, Baheya Foundation for Early Detection and Treatment of Breast Cancer, Giza, Egypt; 2 Department of Radiology, Al Kasr Al-Ainy Hospital, Cairo, Egypt; 3 Department of Radiology, Saudi German Hospital, Cairo, Egypt; 4 Department of Surgery, Baheya Hospital for Early Detection and Treatment of Breast Cancer, Giza, Egypt; 5 Department of Pathology, Baheya Hospital for Early Detection and Treatment of Breast Cancer, Giza, Egypt

**Keywords:** Positron emission mammography, Contrast-enhanced spectral mammography, Breast MRI, Non-mass enhancement, Breast cancer

## Abstract

**Objectives:**

To evaluate the diagnostic performance of positron emission mammography (PEM) as an adjunct to breast MRI or contrast-enhanced spectral mammography (CESM) for characterizing non-mass enhancement (NME).

**Materials and methods:**

This retrospective two-arm study included 108 women with NME. Patients were categorized into Arm 1 (MRI + PEM, *n* = 54) or Arm 2 (CESM + PEM, *n* = 54). Two experienced radiologists independently reviewed all imaging studies blinded to histopathology. Diagnostic performance metrics were calculated using histopathology as the reference standard.

**Results:**

In Arm 1, PEM demonstrated 100% sensitivity (34/34) and higher specificity (100%, 20/20) compared with MRI (30.0%, 6/20; *P* < 0.001). In Arm 2, PEM showed 100% sensitivity (28/28) and higher specificity (92.3%, 24/26) compared with CESM (61.5%, 16/26; *P* < 0.01). Inter-observer agreement was higher for PEM (κ = 0.674) than for MRI (κ = 0.351) and CESM (κ = 0.210). In a combined analysis of 38 indeterminate cases, PEM correctly reclassified 29 of 30 benign lesions.

**Conclusion:**

In this cohort, PEM demonstrated high diagnostic performance and improved specificity when used as an adjunct to MRI or CESM. These findings suggest that PEM may serve as a valuable problem-solving tool in selected indeterminate cases. However, results should be interpreted with caution given the retrospective design and limited sample size.

## Introduction

Non-mass enhancement (NME) of the breast represents one of the most diagnostically challenging interpretations in breast imaging. Defined by the Breast Imaging Reporting and Data System (BI-RADS) lexicon as an area of enhancement not constituting a discrete space-occupying lesion, NME encompasses a wide and histologically diverse spectrum of pathologies [1, 2]. This spectrum ranges from benign entities such as fibrocystic change, adenosis, and stromal fibrosis to high-risk lesions including atypical ductal hyperplasia, and ultimately to malignancies including ductal carcinoma in situ (DCIS) and invasive carcinoma [1, 3]. The inherent ambiguity of NME poses a significant clinical dilemma, as its morphologic patterns on imaging often lack clear-cut features distinguishing benign from malignant disease.

Dynamic contrast-enhanced magnetic resonance imaging (DCE-MRI) is widely regarded as the most sensitive modality for detecting breast NME, capable of revealing lesions occult to mammography and ultrasound [3, 4]. However, this high sensitivity comes at the cost of variable and often modest specificity. The interpretation of MRI is frequently confounded by background parenchymal enhancement (BPE), which can mask or mimic pathology, and by overlapping kinetic curves and enhancement patterns of benign proliferative changes and malignancy [5, 6]. Consequently, radiologists face a high rate of false-positive interpretations, leading to a substantial number of unnecessary biopsies with associated patient anxiety, cost, and procedural risk.

Contrast-enhanced spectral mammography (CESM) has emerged as a promising and more accessible alternative to MRI. By leveraging the differential uptake of an iodinated contrast agent between benign and malignant tissue, CESM provides functional information superimposed on a high-resolution anatomic background [7]. While studies have confirmed its high sensitivity, comparable to MRI, CESM similarly suffers from limited specificity in the context of NME [8]. Benign hormonal stimulation, inflammatory conditions, and other proliferative changes can exhibit pronounced enhancement on CESM, perpetuating the diagnostic challenge and the cycle of unnecessary interventions [7, 8].

Positron emission mammography (PEM) is a high-resolution, breast-dedicated molecular imaging technique that directly addresses this need. By utilizing the radiotracer fluorodeoxyglucose (FDG), PEM maps the regional glucose metabolism of breast tissue [9, 10]. Prior studies have suggested that PEM may offer improved specificity in selected breast imaging settings, likely because it provides metabolic information that is less affected by background parenchymal enhancement and breast density than contrast-based imaging alone [9–11]. Its ability to differentiate metabolically quiescent benign lesions from hypermetabolic cancers provides a powerful, physiologically based contrast mechanism.

However, the specific role of PEM in the diagnostic algorithm for NME—particularly in the most challenging scenarios where first-line modalities yield indeterminate results—remains systematically underexplored. While previous research has established PEM’s value in evaluating suspicious breast lesions in general, its targeted application as a problem-solving tool for indeterminate NME findings has not been comprehensively validated in a dual-arm, retrospective setting. This gap in the literature leaves clinicians without clear guidance on how to integrate this advanced metabolic imaging tool into complex decision-making pathways.

Therefore, the purpose of this retrospective, two-arm study was to evaluate the diagnostic performance of PEM in a cohort of patients with breast NME and to assess its specific value as an adjunctive tool in cases where conventional imaging yielded indeterminate findings.

## Materials and methods

### Study design and population

This was a retrospective, two-arm, single-center study. We searched the institutional database to identify eligible patients who underwent imaging between March 2023 and September 2025. The study received institutional ethics approval with a waiver for informed consent due to its retrospective nature. We identified 108 consecutive women with NME who had undergone both:

- Arm 1 (*n* = 54): Breast MRI followed by PEM.
- Arm 2 (*n* = 54): CESM followed by PEM.

Patient allocation to arms was based on the imaging studies they had received as part of their standard clinical care.

Inclusion criteria were female patients aged ≥ 18 years; presence of NME on archived CESM or MRI images; availability of a subsequent PEM study; and histopathologic verification of the NME lesion.

Exclusion criteria were poor image quality; pregnancy or lactation at time of imaging; prior breast surgery, radiotherapy, or implants in the affected breast; contraindication to iodinated or gadolinium contrast media; and known allergy or uncontrolled diabetes precluding FDG injection.

Because patients were included only if they underwent both conventional contrast-enhanced imaging and subsequent PEM with histopathologic verification, the study cohort reflects a selected diagnostic population rather than unselected screening cohort.

### Imaging protocols

Imaging was performed according to standard institutional clinical protocols at the time of acquisition.

#### MRI acquisition 

MRI examinations were performed using a 1.5-T system with a dedicated bilateral breast coil. The standard protocol included: axial T1-weighted spin-echo sequence (TR/TE: 600/12 ms); Short Tau Inversion Recovery (STIR) sequence for fat suppression; and dynamic contrast-enhanced (DCE) sequence using a 3D fast gradient echo technique (VIBE) acquired before and at 7 post-contrast phases at 60-second intervals. A gadolinium-based contrast agent (0.1 mmol/kg) was administered intravenously at 3 mL/s.

#### CESM acquisition

CESM was performed using a GE Senographe Pristina digital mammography unit with dual-energy acquisition. After intravenous administration of iodinated contrast (1.5 mL/kg) at 3 mL/s, bilateral craniocaudal (CC) and mediolateral oblique (MLO) views were acquired beginning 2 min post-injection.

#### PEM acquisition

PEM was performed using a Naviscan PEM Flex™ Solo II system. Following a fasting period of ≥ 6 h, patients received 370 MBq of ¹⁸F-FDG intravenously. Blood glucose was verified to be < 150 mg/dL prior to injection. Imaging commenced 60 min post-injection, with bilateral CC and MLO views acquired for 10 min each. Quantitative parameters assessed included Maximum Positron Uptake Value (PUVmax) and Lesion-to-background ratio (LTB).

### Timing of imaging and pathologic verification

The interval between the primary imaging examination (MRI or CESM) and PEM, as well as the interval between imaging and histopathologic sampling, was recorded from the medical record. PEM was performed after the primary imaging modality as part of further diagnostic work-up. Histopathologic verification was obtained by core-needle biopsy or surgical excision according to standard clinical management.

### Image interpretation

Two experienced breast radiologists (> 10 years of overall experience and > 5 years with PEM) independently reviewed all archived images in separate sessions, blinded to pathology results and to the other reader’s assessment. They had access to mammography, ultrasound and clinical history.

For MRI and CESM, lesions were categorized according to the BI-RADS assessment system. For the primary analysis, lesions categorized as BI-RADS ≤ 3 were considered benign and lesions categorized as BI-RADS ≥ 4 were considered malignant/suspicious. PEM examinations were interpreted independently and then correlated by lesion location with the corresponding MRI or CESM finding.

Indeterminate cases were predefined as lesions initially categorized as BI-RADS 3–4 A on the primary imaging modality. Discrepancies between readers were resolved by consensus.

When multiple lesions were present, each lesion was analysed separately, with lesion matching based on location and pathology.

### Histopathologic correlation

All lesions had undergone histopathologic verification either by ultrasound-guided or stereotactic core-needle biopsy (5–7 cores using a 14-gauge needle) or following surgical excision. Specimens had been evaluated by a dedicated breast pathologist. Histopathologic analysis included tumour type and grade, receptor status (ER, PR, HER2), and proliferation index (Ki-67%). Lesions were classified as benign, atypical, in-situ carcinoma, or invasive carcinoma accordingly. No high-risk/atypical lesions were included in the final binary diagnostic performance analysis.

### Statistical analysis

All data were analysed using SPSS version 27.0. Analyses were performed separately for each arm. Diagnostic performance metrics (sensitivity, specificity, PPV, NPV, and accuracy) were calculated for each modality with 95% confidence intervals (CI). Inter-observer agreement was assessed using Cohen’s κ coefficient. Receiver operating characteristic (ROC) curves were generated, and areas under the curve (AUC) were compared using the DeLong test within each arm. Subgroup analyses were performed according to breast density (ACR a–d) and background parenchymal enhancement level. A p-value < 0.05 was considered statistically significant. All analyses were performed on a per-lesion basis.

## Results

### Patient and lesion characteristics

The final analysis included 108 women, with 54 in each arm. In Arm 1, histopathology confirmed 34 malignant and 20 benign lesions. In Arm 2, there were 28 malignant and 26 benign lesions. Lesion characteristics were similar between arms (Table 1).

**Table 1:** Patient and lesion characteristics by study arm

Characteristic	Arm 1 (MRI + PEM) *n* = 54	Arm 2 (CESM + PEM) *n* = 54
Mean Age (years)	44 ± 8	45 ± 7
**Final Pathology**		
-Malignant	34 (63%)	28 (51.9%)
- Benign	20 (37%)	26 (48.1%)

### Diagnostic performance by Arm

*Arm 1 (MRI + PEM): * PEM demonstrated 100% sensitivity (34/34) and significantly higher specificity (100%, 20/20) compared to MRI (30.0%, 6/20) (P<0.001). The AUC for PEM (0.95 [95% CI: 0.90, 0.99]) was significantly greater than for MRI (0.78 [95% CI: 0.70, 0.85]) (DeLong test, P<0.01).
*Arm 2 (CESM + PEM): * PEM showed 100% sensitivity (28/28) and significantly higher specificity (92.3%, 24/26) compared to CESM (61.5%, 16/26) (P<0.01). The AUC for PEM (0.92 [95% CI: 0.85, 0.97]) was significantly greater than for CESM (0.75 [95% CI: 0.66, 0.83]) (DeLong test, P<0.01). (Table 2)

**Table 2:** Diagnostic performance by study arm

Arm	Modality	Sensitivity % (*n*)	Specificity % (*n*)	PPV %	NPV %	Accuracy %
1	MRI	100% (34/34)	30% (6/20)	70.8	100	74.1
1	PEM	100% (34/34)	100% (20/20)	100	100	100
2	CESM	89.3% (25/28)	61.5% (16/26)	71.4	84.2	75.9
2	PEM	100% (28/28)	92.3% (24/26)	93.3	100	96.3

### Inter-observer agreement

Inter-observer agreement was substantial for PEM (κ = 0.674) across both arms, significantly higher than for MRI (κ = 0.351) in Arm 1 or CESM (κ = 0.210) in Arm 2.

### Combined analysis of indeterminate cases

Across both arms, 38 cases were considered indeterminate based on the primary modality’s findings. PEM correctly reclassified 29 of 30 benign lesions (96.7% success rate) while maintaining 100% sensitivity for the 8 malignant lesions in this subgroup. This represents a 76.3% reduction in potential false-positive interventions within the indeterminate cohort (Tables 3 and 4).

**Table 3:** Performance summary

Metric	Arm 1 (MRI vs. PEM)	Arm 2 (CESM vs. PEM)
Sensitivity	Equal in this cohort (100% vs. 100%)	Higher for PEM (100% vs. 89.3%)
Specificity	Higher for PEM (100% vs. 30.0%)	Higher for PEM (92.3% vs. 61.5%)
Potentially avoided Biopsies	14 of 15 benign indeterminate cases	15 of 15 benign indeterminate cases

**Table 4:** Performance in indeterminate cases subgroup

Analysis Group	Cases (*n*)	Malignant lesions (*n*)	Benign lesions (*n*)	PEM Correct Benign reclassifications (*n*/*N*)	Avoided biopsies	Sensitivity in malignancy
Arm 1 (MRI) Indeterminate	20	5	15	14/15 (93.3%)	70%	5/5 (100%)
Arm 2 (CESM) Indeterminate	18	3	15	15/15 (100%)	83.3%	3/3 (100%)
Total	38	8	30	29/30 (96.7%)	76.3%	8/8 (100%)

### Subgroup analysis

Exploratory subgroup analyses suggested that PEM performance trends remained favorable across varying breast densities and BPE levels. However, these findings are limited by the modest sample size and should be interpreted as hypothesis-generating only.

## Discussion

**Fig. 1:**
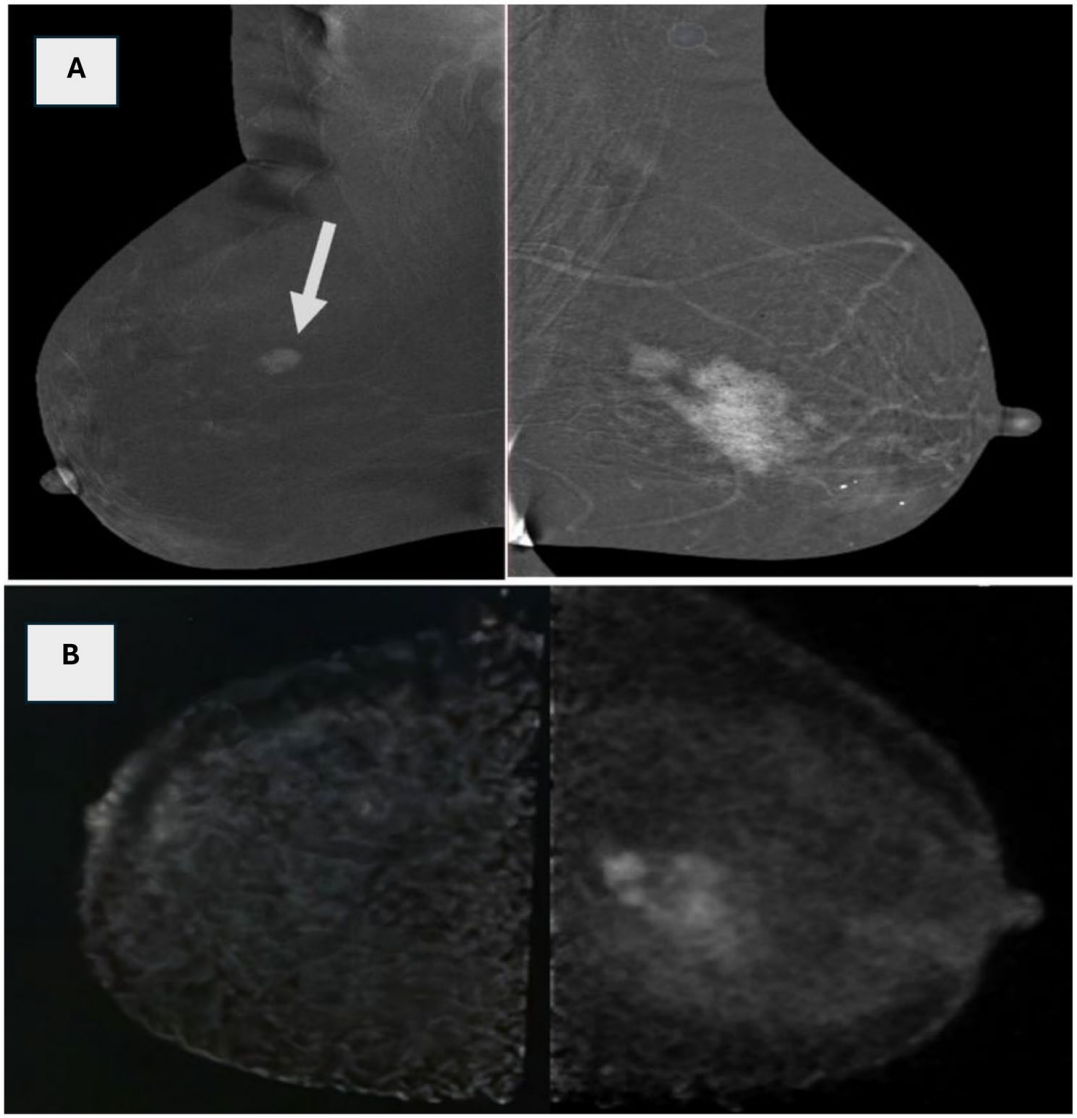
**(A)**: MLO image of CEM demonstrates a left deeply located clumped segmental non-mass enhancement and another right focal NME worrisome for bilateral disease. (**B)**: Corresponding PEM image revealed left breast avid FDG uptake segmental non-mass lesion. Right breast is normal showing no Avid FDG uptake/suspicious lesions. Histopathology; left multifocal locally advanced malignant process. While right lesion proved to be sclerosing adenosis

**Fig. 2:**
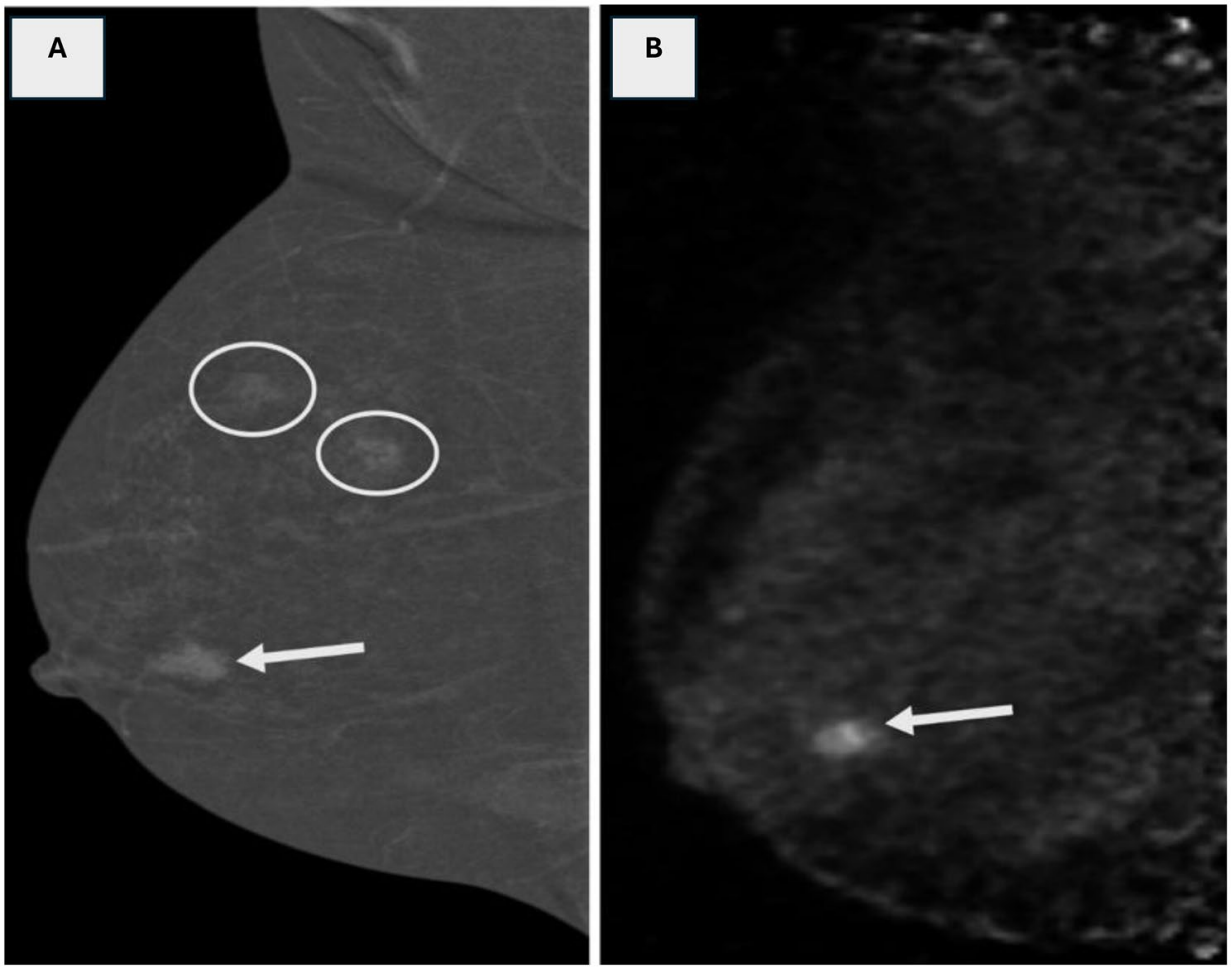
30 years female presenting with a right breast lump. **(A)** MLO CEM image demonstrates multiple enhancing lesions, including a retroareolar mass (arrow) and two superiorly located non-mass enhancements (circles), raising concern for multicentric disease. **(B)** Corresponding MLO PEM image shows focal radiotracer uptake limited to the retroareolar mass (arrow), while the superior non-mass lesions exhibit no significant metabolic activity. **Histopathological analysis confirmed the PEM findings**: the retroareolar lesion was malignant, while the superior non-mass lesions proved to be benign fibrocystic changes. This differentiation was crucial in guiding management, allowing for breast-conserving surgery in a patient initially considered for more extensive resection due to suspected multicentric disease

**Fig. 3:**
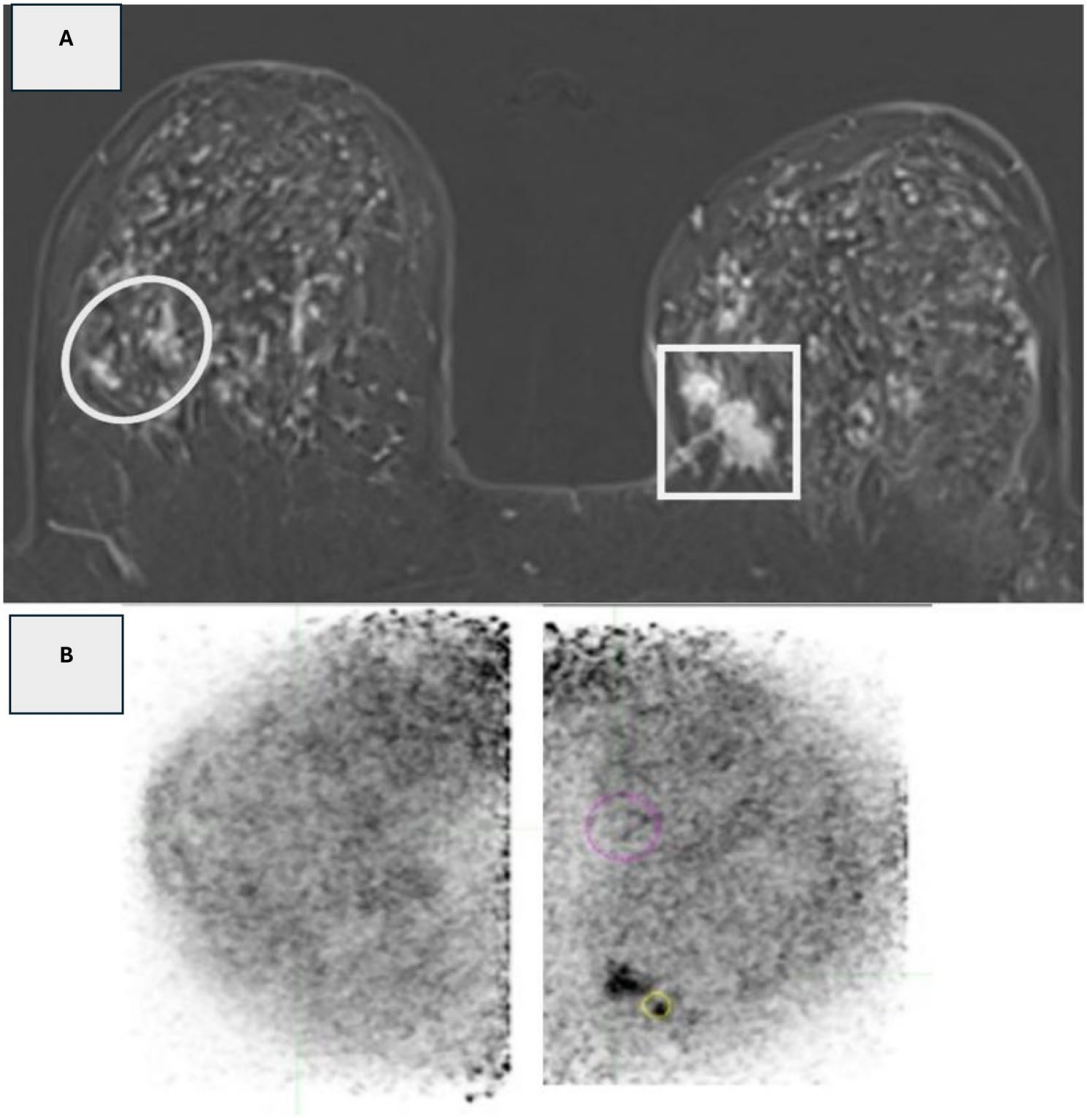
(**A**): Axial T1 post contrast DE-MRI revealed left breast focal heterogeneously enhancing lesions (square), right suspicious non-mass enhancements (circle) as well as bilateral nodular lesions of indeterminate nature. **(B)**: Corresponding PEM image revealed left breast FDG non-mass lesions with PUV max (1.3) and LTB (1.9), No right lesions, and bilateral low FDG nodules probably benign in nature. Histopathology: Left breast lesions proved to be grade-1 tubular carcinoma, and the non-mass lesions at right proved to be fibrocystic changes

**Fig. 4:**
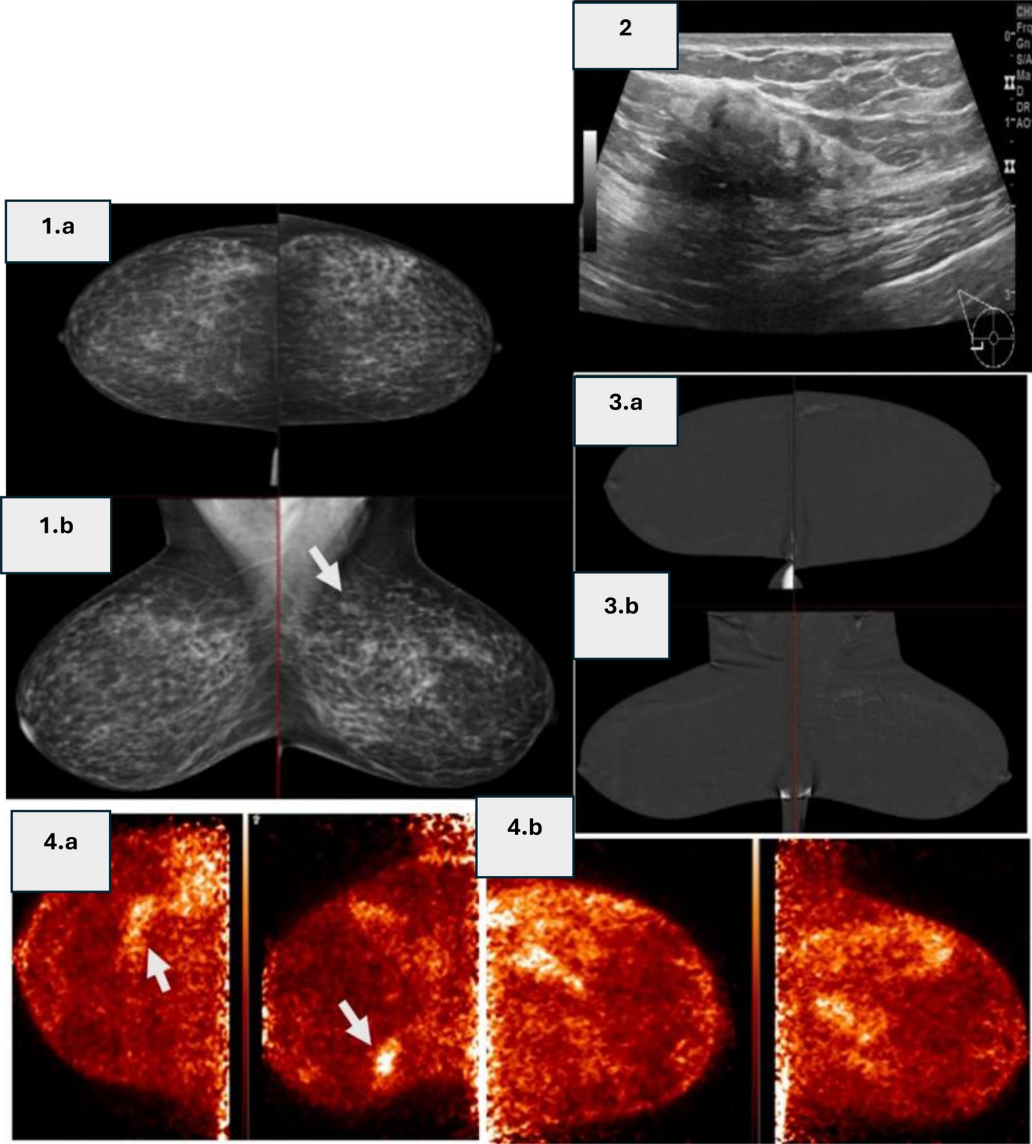
45 years female undergoing screening. (1. **a**) CC and (1. **b**) MLO Digital mammography (DM) show left upper quadrant single view asymmetry(arrow). (2) Grayscale ultrasound image of the right breast in the lower outer quadrant shows a heterogeneous, non-mass lesion with indistinct margins. (3.**a**) CC and (3. **b**) MLO Contrast-enhanced mammography (CEM) demonstrates linear non-mass enhancement in the upper outer quadrant of left breast (arrows), corresponding to asymmetry in DM, which was biopsy-proven for invasive lobular carcinoma, Yet no abnormal enhancing lesions at right side. (4.**a**) Right CC and MLO and (4. **b**) Left CC and MLO Positron emission mammography (PEM) of the same breasts shows corresponding left breast FDG-avidity, as well as FDG-avidity at the right side lower outer quadrant (arrow) corresponding to the abnormal area seen in U/S which warranted further biopsy. Yet, biopsy from the right lesion demonstrated PASH

**Fig. 5:**
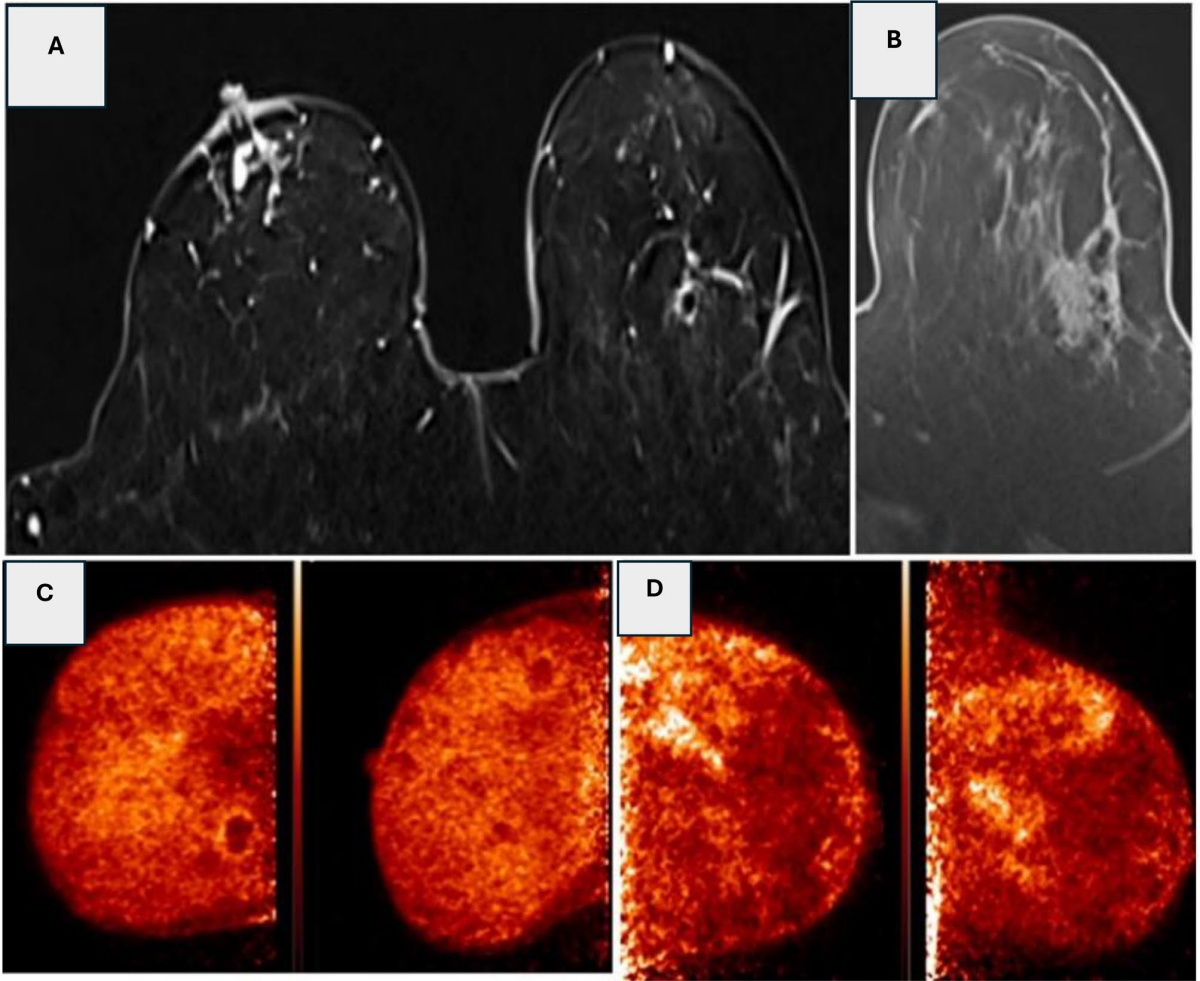
60-year-old female with pathologically proven left invasive lobular carcinoma (ILC) and bilateral pathological axillary lymph nodes. **(A)** axial T2 Fat Supp. and **(B)** axial T1-Post contrast MRI image demonstrates left heterogeneously enhancing segmental NME a right retro areolar NME (arrow) with type-II kinetic curve. The lesion was categorized as indeterminate, and the possibility of bilateral disease could not be excluded. **(C)** Corresponding CC PEM image shows no radiotracer uptake in the right-side region of interest, suggesting a non-metabolically active process. Histopathological analysis confirmed the PEM findings: the right retro areolar lesion was an intra-canalicular fibroadenoma, highlighting PEM’s role in resolving diagnostic uncertainty and avoiding overtreatment

This retrospective, two-arm diagnostic study provides evidence that positron emission mammography (PEM) may enhance the evaluation of diagnostically challenging breast non-mass enhancement. In this cohort, PEM demonstrated high specificity and sensitivity when added to the clinical workflow of either breast MRI or contrast-enhanced spectral mammography. In Arm 1, PEM achieved 100% specificity compared to 30.0% for MRI; in Arm 2, PEM achieved 92.3% specificity compared to 61.5% for CESM. Furthermore, PEM exhibited substantially higher inter-observer agreement compared to conventional modalities. In a pre-specified analysis of indeterminate cases, PEM functioned as an effective problem-solving tool, correctly reclassifying 29 of 30 benign lesions and demonstrating potential to reduce unnecessary biopsies in this challenging subgroup (Figs. 1, 2, 3, 4 and 5).

The persistently high false-positive rate associated with NME on contrast-enhanced imaging represents a significant clinical and economic burden in breast radiology. Both DCE-MRI and CESM rely on the detection of angiogenesis and increased vascular permeability, physiological features that are not exclusive to malignancy but are also hallmarks of a wide spectrum of benign proliferative and inflammatory conditions [1, 3, 6]. This fundamental biological overlap is the root cause of the modest specificities observed for MRI and CESM in their respective arms, figures that are consistent with prior literature reporting specificities of 30–65% for NME characterization [1, 8]. The diagnostic dilemma is particularly pronounced in premenopausal women or those on hormone replacement therapy, where background parenchymal enhancement can obscure or mimic pathology, leading to interpretive uncertainty and a low threshold for biopsy [5, 6].

The higher specificity of PEM observed in this study is intrinsically linked to its fundamental imaging mechanism. Unlike MRI and CESM, which image secondary effects of tumorigenesis, PEM directly maps cellular glucose metabolism via the uptake of ¹⁸F-FDG. Malignant cells typically exhibit a glycolytic phenotype, leading to markedly increased FDG avidity [9, 10]. This may provide a more specific biological contrast between malignant and benign tissue. In our study, this translated into an ability to distinguish hypermetabolic cancers from metabolically quiescent benign entities such as fibrocystic changes, adenosis, and stromal fibrosis, which constituted the majority of our benign cases. The two false-positive PEM cases in Arm 2 corresponded to pseudoangiomatous stromal hyperplasia (PASH) lesions, underscoring that benign proliferative breast lesions may occasionally demonstrate FDG uptake and yield false-positive findings on metabolic imaging [12].

A particularly compelling finding of our study is the significantly higher inter-observer agreement achieved with PEM (κ = 0.674) compared to both MRI (κ = 0.351) and CESM (κ = 0.210). The interpretation of NME on contrast-enhanced modalities is notoriously subjective, requiring the synthesis of complex and sometimes subtle morphologic patterns and kinetic curves. This subjectivity inevitably leads to variability in BI-RADS assessment. PEM, by contrast, provides a quantitative and visually stark metric of disease—focal radiotracer uptake—that is less susceptible to interpretive variance. This reproducibility is a critical advantage in clinical practice, as it promotes standardized reporting and enhances referring clinicians’ confidence in imaging recommendations.

The clinical utility of any new diagnostic test is ultimately measured by its impact on patient management (Fig. 2). Our analysis of the 38 indeterminate cases across both arms provides the most direct evidence for PEM’s potential value as a problem-solving tool. In these scenarios, where the primary modality yielded equivocal or suspicious findings, the adjunctive metabolic data from PEM enabled confident downgrading of 29 out of 30 benign lesions. This 96.7% success rate in benign lesion reclassification could prevent 76% of unnecessary biopsies in this specific subgroup. In this cohort, adjunctive PEM downgraded many benign indeterminate lesions without missing malignancy; however, this observation requires confirmation in larger prospective studies before being generalized to broader practice.

### Limitations and considerations

Several important limitations must be acknowledged when interpreting these findings.

First, the very high diagnostic performance observed for PEM, particularly in Arm 1, may partly reflect the retrospective case selection process, which likely enriched the cohort for lesions referred for further problem-solving evaluation after equivocal conventional imaging findings. Accordingly, these results should not be interpreted as representative of a general screening or unselected diagnostic population.

Second, this study is subject to significant work-up (verification) bias inherent in its retrospective, single-center design. Only patients with suspicious NME on MRI/CESM who were subsequently referred for PEM and had histopathologic confirmation were included. This selection process artificially enriches the cohort for malignancy and diagnostically challenging benign findings, thereby inflating positive predictive value and limiting generalizability. Consequently, the diagnostic accuracy metrics reported here apply strictly to a problem-solving context.

Third, radiation exposure represents a major consideration for PEM. The administered activity of 370 MBq (10 mCi) of ¹⁸F-FDG used in this study results in an estimated effective dose of approximately 6–8 mSv. This dose is higher than mammography and absent in MRI. Therefore, the use of PEM in this setting should adhere to the ALARA principle, reserved specifically for scenarios where the avoidance of an unnecessary biopsy in a metabolically quiescent lesion outweighs the stochastic radiation risk. This is particularly relevant in younger patients where lifetime attributable risk is greater.

Fourth, PEM is not widely available and is associated with higher costs compared to conventional modalities. It should be viewed as a niche, targeted adjunct rather than a routine diagnostic test.

Fifth, this study did not include a direct comparison with PET/CT or PET/MRI, which limits broader contextualization of these findings [11]. PEM provides breast-dedicated metabolic imaging with high spatial resolution (~ 1.5–2 mm). PEM may offer improved detection and characterization of small or subtle lesions, such as non-mass enhancement, whereas conventional PET/CT (~ 4–6 mm) and PET/MRI are primarily designed for whole-body staging rather than detailed breast lesion characterization [9, 11]. Future studies comparing these modalities directly are warranted.

Finally, the modest sample size (*n* = 108) limits the robustness of subgroup analyses, which should be interpreted as exploratory trends only. Prospective validation in a larger, more generalizable population is required.

### Clinical relevance statement

Non-mass enhancement (NME) remains a diagnostic challenge, with conventional contrast-enhanced imaging limited by poor specificity. This retrospective, dual-arm study demonstrates that positron emission mammography (PEM), when used as an adjunct to MRI or CESM, provides additional metabolic information that may improve the characterization of indeterminate NME. Its selective use as a problem-solving tool may help reduce unnecessary biopsies while maintaining diagnostic confidence.

## Conclusion

In this cohort, PEM demonstrated high diagnostic performance and improved specificity when used as an adjunct to MRI or CESM for evaluating NME. These findings suggest that PEM may serve as a targeted problem-solving tool in selected indeterminate cases, with the potential to reduce unnecessary biopsies. However, results should be interpreted with caution given the retrospective design, potential selection bias, and limited sample size. Further prospective studies are required to validate these observations and define the optimal clinical niche for PEM in breast imaging.

## Data Availability

The datasets analyzed during the current study are not publicly available due to institutional and patient confidentiality restrictions but are available from the corresponding author on reasonable request, subject to institutional approval.

## Abbreviations

ACR: American College of Radiology
AUC: Area Under the Curve
BPE: Background Parenchymal Enhancement
CC: Craniocaudal
CESM: Contrast-Enhanced Spectral Mammography
CI: Confidence Interval
DCIS: Ductal Carcinoma in situ
DCE-MRI: Dynamic Contrast-Enhanced Magnetic Resonance Imaging
ER: Estrogen Receptor
FDG: Fluorodeoxyglucose
HER2: Human Epidermal Growth Factor Receptor 2
LTB: Lesion-to-Background Ratio
MLO: Mediolateral Oblique
MRI: Magnetic Resonance Imaging
NME: Non-Mass Enhancement
NPV: Negative Predictive Value
PASH: Pseudoangiomatous Stromal Hyperplasia
PEM: Positron Emission Mammography
PPV: Positive Predictive Value
PR: Progesterone Receptor
PUV: Positron Uptake Value
ROC: Receiver Operating Characteristic
STIR: Short Tau Inversion Recovery
TR/TE: Repetition Time/Echo Time
VIBE: Volumetric Interpolated Breath-hold Examination
